# Smartphone use habits of anesthesia providers during anesthetized patient care: a survey from Turkey

**DOI:** 10.1186/s12871-016-0245-7

**Published:** 2016-10-06

**Authors:** Hüseyin Ulaş Pınar, Omer Karaca, Rafi Doğan, Ümmü Mine Konuk

**Affiliations:** Anesthesiology Department, Konya Research Center, Baskent University, Hocacihan Mah. Saray Cad. No: 1, Selçuklu, Konya 42080 Turkey

**Keywords:** Smartphone, Anesthesia, Anesthesia providers

## Abstract

**Background:**

Smartphones are used in many areas of anesthesia practice. However, recent editorial articles have expressed concerns about smartphone uses in the operating room for non-medical purposes. We performed a survey to learn about the smartphone use habits and views of Turkish anesthesia providers.

**Methods:**

A questionnaire consisting of 14 questions about smartphone use habits during anesthesia care was sent anesthesia providers.

**Results:**

In November-December 2015, a total of 955 participants answered our survey with 93.7 % of respondents responding that they used smartphones during the anesthetized patient care. Phone calls (65.4 %), messaging (46.4 %), social media (35.3 %), and surfing the internet (33.7 %) were the most common purposes. However, 96.7 % of respondents indicated that smartphones were either never or seldom used during critical stages of anesthesia. Most respondents (87.3 %) stated that they were never distracted because of smartphone use; however, 41 % had witnessed their collagues in such a situation at least once.

**Conclusions:**

According to the results of the survey, smartphones are used in the operating room often for non-medical purposes. Distraction remains a concern but evidence-based data on whether restrictions to smartphone use are required are not yet available.

## Background

Smartphone use has recently undergone large-scale increases worldwide, and smartphones have become an indispensable part of daily life. These small, handy technological devices have provided unique opportunities for use in medicine, as in many other aspects of life. In the developing world, smartphones may be used for anesthesiology practice including: team member communication [1], knowledge acquisition through internet or downloaded applications, information transfer, e-learning, telemedicine or remote monitoring [2]. Papers have recently been published on the use of smartphones as oximetry devices [3] or stethoscopes [4], for determining neuromuscular function [5], maintaining 15° of left lateral tilt during caesarean section [6], fiberoptic bronchoscopy education [7], pain scoring [8], and diagnosing arrhythmia/dysrhythmia [9].

Concurrently with various publications on smartphones uses in anesthesiology practice, some editorials have also expressed concerns about non-medical smartphone/laptop use by anesthesia providers during anesthetized patient care [10–12]. This subject has drawn media attention since an anesthesiologist was charged with distraction due to smartphone or iPad use after the death of a patient during AV node ablation in Dallas, Texas, USA in 2011 [13].

As yet there is no clear answer to the question of whether smartphones are distracting devices or useful communication devices. For instance, is it safe to use smartphones or laptops to read about anesthesiology or perform Pubmed or e-library literature searches during patient monitoring? Worldwide restriction policies by associations like the American College of Surgeons and the American Association of Nurse Anesthesists have instituted warnings that smartphones are a distracting factor especially in the operating room but they do not suggest full restriction of smartphone usage in operating rooms [14, 15]. Despite concerns about smartphone use, no study has yet evaluated the impact of smartphone use on anesthesiologists. To make performance assessments for anesthesiology, it is necessary to focus on the domain of anesthesia practice. Monitoring anesthetized patients is a job that requires multitasking and maintenance of situational awareness [10]. Anesthesiologists perform monitor checks at short intervals, which occupy 5 % of their overall time [16]. Smartphone use may occupy some of the rest.

In this questionnaire study, we determined the descriptive characteristics of smartphone use by anesthesia providers during monitoring of anesthetized patients in Turkey.

## Methods

This study was approved by the Baskent University Institutional Review Board (Project No: KA 15/366; Chairman: Prof. Dr. Hakan Özkardeş) and supported by the Baskent University Research Fund. During November 2015_December 2015, a 14-item questionnaire created on docs.google.com was distributed to a group of certified anesthesia nurses and anaesthesia residents by sending its electronic link to their e-mail groups; the questionnaire was also handed out in written form at the national meeting of the Turkish Society of Anesthesia and Reanimation held during the same period. This questionnaire was based on a study by Smith et al. [17] that explored cell phone use by perfusionists during cardiopulmonary bypass. All of the questions in the questionnaire were multiple-choice. The first-third parts of the questionnaire assessed demographic properties, frequency and purpose of smartphone use during anesthetized patient care, and respondents’ opinions on for smartphone usage during patient care (Appendix). Statistical analysis between age groups for smartphone use during patient care was performed via Chi-square test using the SPSS (Statistical Package for Social Science) for Windows 11.5 software package. The results were considered statistically significant when *p* <0.05.

## Results

### Demographic data

The questionnaire was completed by 955 subjects; response rate was 22 % for the e-mail questionnaire (600 responses) and 19 % for the one handed out at the national meeting (355 responses). Of these, 325 (34 %) were anesthesia nurses, 311 (32.5 %) were anesthesia residents, 251 (26.2 %) were senior anesthesia physicians, and 68 (7.1 %) were faculty members in anesthesiology. Because 542 participants (56.9 %) were aged 20_30 years, and 283 (29.7 %) were aged 31_40 years, the majority of the participants were from younger age groups. The age range of 116 (12.2 %) participants was 41_50 years, and 9 (0.9 %) were aged 51_60 years; only three were aged >60 years. Two hundred ninety-five (31.1 %) participants were working at state hospitals, 289 (30.5 %) at university hospitals, 189 (19.8 %) at private hospitals, and 174 (18.3 %) at state training and research hospitals. Nine hundred thirty-seven (98.5 %) participants owned smartphones. There was no restriction on smartphone use in the operating room at 73.6 % of participants’ institutions; 19.2 % of participants were allowed only in-house smartphone communication, and 7.1 % of respondents were fully restricted from smartphone use in the operating room.

### Smartphone use

**Fig. 1:**
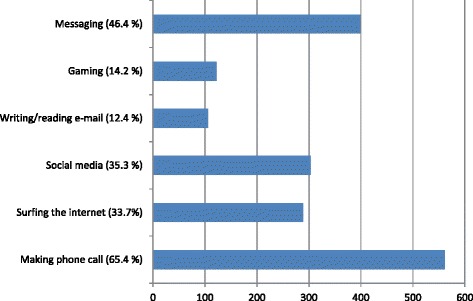
Purposes of smartphone use (shown as respondent numbers and percentages in parantheses)

**Fig. 2:**
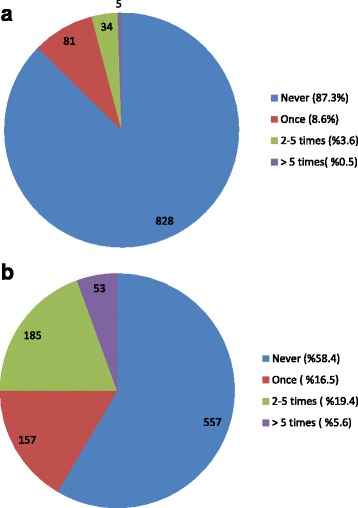
The responses of the participants to the question whether (**a**) themselves or (**b**) another anaesthesia provider has ever been distracted with smartphone use. (shown as percentages in parantheses and respondent number on graphic)

**Fig. 3:**
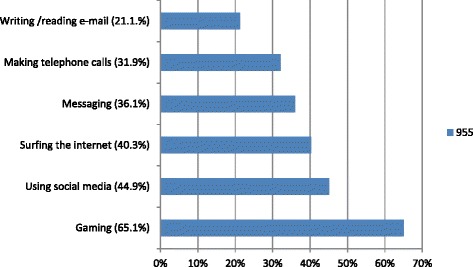
Which form of smartphone usage do you think would distract users or produce a negative impact? (The participants were allowed to select more than one option.) (Shown as percentages in parentheses)

93.7 % of respondents used smartphones during anesthetized patient care. The frequency of smartphone use is presented by age in Table 1. There is no difference between the 20_30-year-old and 31–40-year-old groups, but there is a significant decrease between participants aged >40 years and younger participants in terms of smartphone use frequency during patient care (*p* = 0.011). Figure 1 shows participants’ purposes of smartphone use, and Table 2 shows their age distribution. The rate of smartphone use was remarked lower at critical stages, such as anesthesia induction and emergence from anesthesia: 77 % of participants (741 respondents) answered that they never use smartphones during those stages. Figure 2 depicts responses to the question about whether the participants had ever experienced distraction or observed it in other anesthesia providers. Figure 3 depicts participants’ opinions about which patterns of smartphone use might lead to distraction or negative medical consequences. Of the respondents, 781 (81.2 %) stated that they had never been warned by surgical the team or their collagues because of their smartphone use, 12.1 % had been warned only once, 4.8 % had been warned 2–5 times, and 2 % had been warned ≥5 times. Restrictions on smartphone use in the operating room were supported by only 72 (7.6 %) of participants whereas; 448 (47 %) believed that smartphone use should not be restricted at all, and 432 (45.3 %) suggested that these devices should be used for in-house communication only. Lastly finally, 498 (52.6 %) of the participants reported that their smartphones contained at least one anesthesia-related application.

**Table 1:** Age-based distribution of smartphone use frequency

	Smartphone use frequency					Total
Age (year)	Never	Seldom	Sometimes	Often	Very often	
20–30	42	132	255	73	37	539
	7,8 %	24,5 %	47,3 %	13,5 %	6,9 %	100,0 %
31–40	15	78	123	46	21	283
	5,3 %	27,6 %	43,5 %	16,3 %	7,4 %	100,0 %
>40	3	50	53	10	11	127
	2,4 %	39,4 %	41,7 %	7,9 %	8,7 %	100,0 %
TOTAL	60	260	431	129	69	949
	6,3 %	27,4 %	45,4 %	13,6 %	7,3 %	100,0 %

χ^2^ = 19,813, *p* = 0,011

Datas are shown as respondent numbers and percentages in groups

**Table 2:** Age-based distribution of purposes of smartphone use during anesthetized patient care (participants were allowed to select more than one option)

Age (years)	Making telephone calls	Internet surfing	Using social media	Writing/reading e-mail	Gaming	Messaging	Total
20–30	259	173	211	60	90	262	473
	54.8 %	36.6 %	44.6 %	12.7 %	19.0 %	55.4 %	
31–40	198	95	76	33	22	101	262
	75.6 %	36.3 %	29.0 %	12.6 %	8.4 %	38.5 %	
41–50	93	18	14	11	9	31	110
	84.5 %	16.4 %	12.7 %	10.0 %	8.2 %	28.2 %	
51–60	5	2	2	2	0	3	6
	83.3 %	33.3 %	33.3 %	33.3 %	0 %	50.0 %	
>60	3	0	0	0	0	0	3
	100.0 %	0 %	0 %	0 %	0 %	0 %	
Total	558	288	303	106	121	397	854

Datas are shown as number of respondents and percentages in groups

## Discussion

By excluding the reply “I never use a smartphone” we determined that the rate of smartphone use in operating room among study participants was 93.7 %. As expected, the most common purposes for smartphone use were phone calls and messaging, with rates of 65.4 and 46.4 %, respectively. Such highsmartphone usage rates may be explained by use for in-house communication. As the majority of the participants were young anesthesia providers, computing use including social media (35.3 %) and surfing the internet (33.7 %) were also among the most common smartphone use purposes in our population. This is the first survey of its kind in anesthesia providers, but similar studies have been undertaken in other healthcare professionals. In 2010, the rate of cell phone use during cardiopulmonary bypass procedures was found to be 55.6 % among perfusionists, although usage rates for purposes other than phone calls and messaging were much lower [17]. Cho et al. reported that smartphone usage rates in clinical practice by nursing students was 46.2 and 24.7 % of them had been distracted by smartphones during clinical practice. According to that study, 83.7 % of nurses had observed someone else distracted by smartphone usage in clinical practice [18]. In 2015, Mcbride et al. reported that 78.1 % of participating nurses used smartphones at work, and they used smartphone; 38.6 % for writing/reading e-mail, 25.7 % to read news, 20.8 % social media, and 6.5 % for playing games [19]. Experienced anesthesiologists have an increased, albeit limited, capability to perform multipl simultaneous tasks, which is more difficult for trainees or less experienced anesthesiologists [10]. Hence, inexperienced anesthesiologists should avoid additional distractors in the operating room, such as smartphone uses. However, our results show that younger anesthesia providers use smartphones more than older providers.

In this study, 87.3 % of the participants stated that they had never experienced any negative medical consequences smartphone use. However, 41 % had witnessed their collagues in such a situation at least once. This discrepancy may be explained by smartphone users’ inability to notice negative medical issues caused by their own smartphone use. In addition, people are known to provide self-protecting answers to self-report questionnaires. Moreover, the vagueness of the term “negative medical issues” may have affected the way the participants replied to the question. The response to the question “which smartphone use applications do you think distracts anesthesia providers?” was most commonly games/gaming (65.1 %), followed by social media (44.9 %) and internet surfing (40.3 %), with telephone calls and messaging having lower rates. Although there is no direct evidence for this assertion, respondents may think gaming is the most distracting factor because it requires continuous attention during play. The rate of smartphone use for computing purposes unrelated to patient care was expectedly high. Only 8.4 % of the participants stated that smartphone use would not have any negative effects. Despite this, only 7.6 % advocated complete restriction of smartphone use, while 45.3 % preferred that smartphone use be limited to in-house calls. In-house smartphone use was completely restricted in 7.1 % of the participants’ institutions.

There is no strong evidence as to whether internet use negatively impacts attention and patient care. An examination of ASA closed claims indicated that 13 of 5822 intraoperative adverse events were caused by operating room distractions. The reasons for distraction included reading written material, fixed voice calls, and listening to high-volume music but they did not include laptop, smartphone, or internet use [12]. However, it would be not surprising if we were to encounter such claims in the near future.

In 2009, Slagle et al. defined intraoperative anesthesia care as “hours of boredom punctuated by moments of terror”, similarly to aviation. They examined the impact of reading on vigilance, workload, and task distribution during patient care. They concluded that anesthesia providers tend to read at times of lower workload and that their vigilance was not impaired during such reading periods [20]. However, because reading is considered as a passive act, smartphone use may be considered even more distracting during social media use, messaging, or gameplay. An investigation similar to the reading study conducted by Slagle et al. could also be designed for smartphone use in the operating room. In a similar study, the impact of the use of non-record-keeping Anesthesia Information Management System workstations on hemodynamic variability and aberrancies was explored. Anesthesia providers spent a significant proportion of their time using non-record-keeping Anesthesia Information Management System workstations, but that did not cause any significant hemodynamic variability or aberrancy in anesthetized patients during general surgery or gynecological procedures [21].

Smartphone use leads to longer reaction time, reduced focud and lowered behavioral performance during cognitive tasks especially driving [22]. Jorm and Roper deconstructed some theoretical smartphone-related errors using safety methodologies and concluded that smartphone use may sometimes detract from the safety of a currently attended patient in order to allow an anesthesiologist to organize the safety of other patients or deals with other work [23]. In the operating room, there are many possible distracting factors in anesthesia practice, which may compromise patient safety. In an observational study Campbell et al. observed anesthesiologists during active patient care and revealed that many distractors during procedures originated from other staff, the working area, external team members, equipment, ambient noise, and the anesthesiologists themselves, with a considerably high average number of distracting events [24]. Distracting events reached an occurrence rate as high as 0.5/min during emergence from anesthesia [24]. Broom et al. examined critical stages of anesthesia and found out that noise and auditory and physical distractors existed at every stage, with noise being more intense during awakening after anesthesia, possibly due to a greater personnel entrance, exit and movements during that period. Those results suggested that the sterile cockpit rules used in aviation for critical phases (under 10.000 ft, landing, and take-off), in which unnecessary conversation and activity are reduced, could also be employ in anesthesiology [25]. Our questionnaire’s results, suggest that smartphone use rates at the intubation and emergence stages were at very low levels.

The results of a 2010 English questionnaire-based study involving 918 subjects, indicated that 80 % of users’, smartphones contained medical applications, 60 % of which were anesthesia applications [26]. Payne et al. reported that nearly 80 % of junior doctors and medical students have at least one medical application on their smartphones [27]. In our study, on the other hand only 52.6 % of users had at least one anesthesia-related medical application on their smartphones. This rate is acceptable for a developing country. Nevertheless, anesthesia-related applications have unknown usage frequencies, because participants were not questionned on the usage details of their existing applications.

This study had some limitations. One is related to the under-representation of older anesthesiologists, because young anesthesiologists constituted the major proportion of the study participants. Moreover, the participants were not asked whether their rates of smartphone use changed according the type of surgery or anesthesia (major vs. minor surgery, regional vs. general anesthesia;). Further, the participants did not provide the details of the medical problems they faced. In addition, self-report questainnares have limitations due to respondent honesty, introspective ability and misunderstanding of questions, thus answers can be deceptive [28, 29].

## Conclusion

In conclusion, many distracting factors put strain on anesthesiologists during patient care. Smartphones with their increasing current trends of use, have also started to emerge as a significant distractor. Their use by Turkish anesthesia providers was ubiquitous. However, because little evidence-based information exists, it appears impractical and potentially unwise to completely restrict the use of an important means of communication that also plays a fundamental role in accessing medical information.

## Appendix

Survey

1. Which is your age range?
  - 20–30
  - 31–40
  - 41–50
  - 51–60
  - >60 ages
2. What is your profession group?
  - Nurse Anesthetist
  - Anesthesia Resident
  - Senior Anesthesia Physicians
  - Faculty Member in Anesthesiology
3. What kind of hospital do you work for?
  - University Hospital
  - State Training and Research Hospital
  - State Hospital
  - Private Hospital
4. Do you have a smartphone?
  - Yes
  - No
5. Is there any restriction for smartphone usage in operating theater at your institution?
  - Yes
  - No
  - Partly (Only in-house communication permitted)
6. How often do you use your smartphone during anesthetized patient care?
  - Very often
  - Often
  - Sometimes
  - Seldom
  - Never
7. For what purposes do you use your smartphone during anesthetized patient care? (you can choose more than one option)
  - Phone calls
  - Surfing internet
  - Social media
  - Writing/reading e-mail
  - Playing games
  - Message (e.g. SMS, Whatsapp)
8. How often do you use your smartphone at critical stages of anesthesia like induction and emergence?
  - Very often
  - Often
  - Sometimes
  - Seldom
  - Never
9. Have you ever experienced any distraction or negative medical consequence because of smartphone usage during anesthetized patient care?
  - Never
  - Once
  - 2–5 times
  - More than 5 times
10. Have you ever witnessed one of your collagues experienced any distraction or negative medical consequence because of smartphone usage during anesthetized patient care?
  - Never
  - Once
  - 2–5 times
  - More than 5 times
11. Which of the following smartphone usage methods might result a distraction or negative medical consequence during anesthetized patient care? (you can choose more than one option)
  - Phone calls
  - Surfing internet
  - Social media
  - Writing/reading e-mail
  - Playing games
  - Message (SMS, whatsapp)
  - None
12. Do you think the smartphone usage should be restricted in operating theaters?
  - Yes, it should be restricted
  - No need for restriction
  - It should be partly restricted (only in-house communication should be allowed)
13. Have you ever been warned by your collagues or surgical team because of smartphone usage during the patient care?
  - No
  - Yes, once
  - Yes, 2–5 times
  - Yes, more than 5 times
14. Is there any anesthesia related application on your smartphone?
  - Yes
  - No
